# Identification of public neoantigens with broad HLA class I coverage as candidates for off-the-shelf cancer vaccine development in colorectal cancer

**DOI:** 10.3389/fimmu.2026.1686224

**Published:** 2026-02-18

**Authors:** Diem TP Tran, Bui Que Tran Nguyen, Huu Thinh Nguyen, Thi Mong Quynh Pham, Dinh Viet Linh Nguyen, Thanh Nhan Nguyen, Thi Kim Cuong Ho, Vy Nguyen, Pham Trung Dung Nguyen, Duc Huy Tran, Thanh Sang Tran, Truong-Vinh Ngoc Pham, Minh-Triet Le, Hoa Giang, Hoai Nghia Nguyen, Minh-Duy Phan, Le Son Tran

**Affiliations:** 1 Department of Immunotherapy, Medical Genetics Institute, Ho Chi Minh City, Vietnam; 2 Department of Genetics, Faculty of Biology and Biotechnology, University of Science, Viet Nam national University Ho Chi Minh city (VNUHCM), Ho Chi Minh City, Vietnam; 3 Vietnam National University, Ho Chi Minh City, Vietnam; 4 University Medical Center Ho Chi Minh (HCM), Ho Chi Minh City, Vietnam

**Keywords:** colorectal cancer (CRC), immunotherapy, off-the-shelf neoantigens, shared neoantigens, vaccine

## Abstract

**Introduction:**

Shared neoantigens derived from recurrent mutations represent promising targets for off-the-shelf (OTS) cancer immunotherapies; however, their clinical utility is often constrained by population-specific HLA diversity and variable immunogenicity. There remains a need for population-tailored neoantigen panels with broad HLA coverage and functional validation.

**Methods:**

We developed a colorectal cancer (CRC)–specific OTS neoantigen panel by integrating TCGA mutation data with HLA class I binding predictions across 68 alleles (18 HLA-A, 34 HLA-B, and 16 HLA-C), covering both Asian and Caucasian populations. Candidate neoantigens were evaluated in a Vietnamese CRC cohort (n = 67) based on mutation and HLA matching. Functional immunogenicity was assessed in patient-derived CD8⁺ T cells using IFN-γ ELISpot assays and single-cell RNA sequencing.

**Results:**

The resulting panel comprised 73 recurrent nonsynonymous mutations, each generating at least one predicted HLA-I–restricted neoepitope, achieving >90% population coverage in both Asian and Caucasian groups. In the Vietnamese CRC cohort, 58% of patients harbored at least one matched neoantigen, with mutation frequency strongly correlating with predicted HLA presentation, particularly for HLA-A alleles. Functional validation in seven patients demonstrated robust CD8^+^ T-cell responses against TP53_R273H. Neoantigen-reactive T cells exhibited transcriptional signatures consistent with cytotoxic effector function and stem-like memory phenotypes.

**Discussion:**

These findings identify TP53_R273H as a clinically relevant shared neoantigen in CRC and support the translational feasibility of a population-tailored OTS neoantigen panel. Our integrative computational and experimental framework highlights the importance of combining population-level HLA coverage with functional validation to advance broadly applicable neoantigen-based immunotherapies.

## Introduction

Colorectal cancer (CRC) is a leading cause of cancer-related morbidity and mortality worldwide, with limited treatment options for advanced-stage disease (1). Although immune checkpoint inhibitors (ICIs) have transformed cancer therapy, their clinical efficacy in CRC is largely confined to microsatellite instability-high (MSI-H) tumors, which account for only ~15% of cases (2). The remaining ~85% of CRCs are microsatellite stable (MSS) and respond poorly to ICIs, primarily due to a low tumor mutational burden (TMB) and limited tumor immunogenicity (3). These challenges underscore the urgent need for alternative immune-based strategies capable of eliciting robust and durable anti-tumor responses in MSS-CRC.

Neoantigens, which are peptides derived from tumor-specific somatic mutations, have emerged as promising targets for immunotherapy because they can elicit highly specific T-cell responses while minimizing off-target toxicity (4). However, personalized neoantigen-based therapies face major challenges—including high costs, lengthy development timelines, and considerable patient-to-patient variability—that limit their clinical feasibility, particularly in low-resource settings such as Vietnam (5). To address these limitations, an alternative strategy is to develop a shared neoantigen panel composed of recurrent, immunogenic mutations commonly found across multiple CRC patients. This approach could facilitate the development of off-the-shelf immunotherapies - such as neoantigen vaccines or T-cell receptor (TCR)-based treatments -with broader accessibility and impact (6). The feasibility of shared neoantigen-based therapies has been demonstrated in other cancers, including melanoma and non-small cell lung cancer, where shared neoantigens have been successfully targeted with cancer vaccines and T-cell therapies (7). In CRC, the NOUS-209 vaccine, an off-the-shelf neoantigen therapy targeting 209 shared mutations in MSI-H tumors, has shown promising clinical outcomes (8). However, no equivalent shared neoantigen strategy currently exists for MSS-CRC, highlighting a significant gap in neoantigen immunotherapy research.

Historically, efforts to identify shared neoantigens in CRC have concentrated on hotspot mutations in key driver genes such as *KRAS* (e.g., G12D, G12V, G13D), *TP53* (e.g., R175H, R273C, Y220C), and *APC*, due to their high prevalence in tumors (9). However, neoantigen panels developed by previous studies—including the TESLA consortium (10)-have largely relied on *in silico* predictions restricted to a limited subset of HLA alleles, primarily HLA-A*02:01. This approach overlooks the vast diversity of HLA genotypes across global populations and results in the selection of neoantigens with predicted binding affinity only for a narrow HLA spectrum. Consequently, these panels may have limited clinical utility in populations where such alleles are less common, such as in East and Southeast Asia. Moreover, by relying on a constrained HLA set, these panels may not sufficiently account for immune escape mechanisms like HLA loss of heterozygosity (LOH), in which tumor cells lose expression of key HLA alleles (11, 12). As a result, the predicted neoantigens may fail to elicit robust T cell responses in diverse patient groups or may be completely missed due to antigen presentation loss. These limitations highlight the need for more inclusive neoantigen discovery strategies that incorporate a broader and population-representative range of HLA alleles.

**Figure 1:**
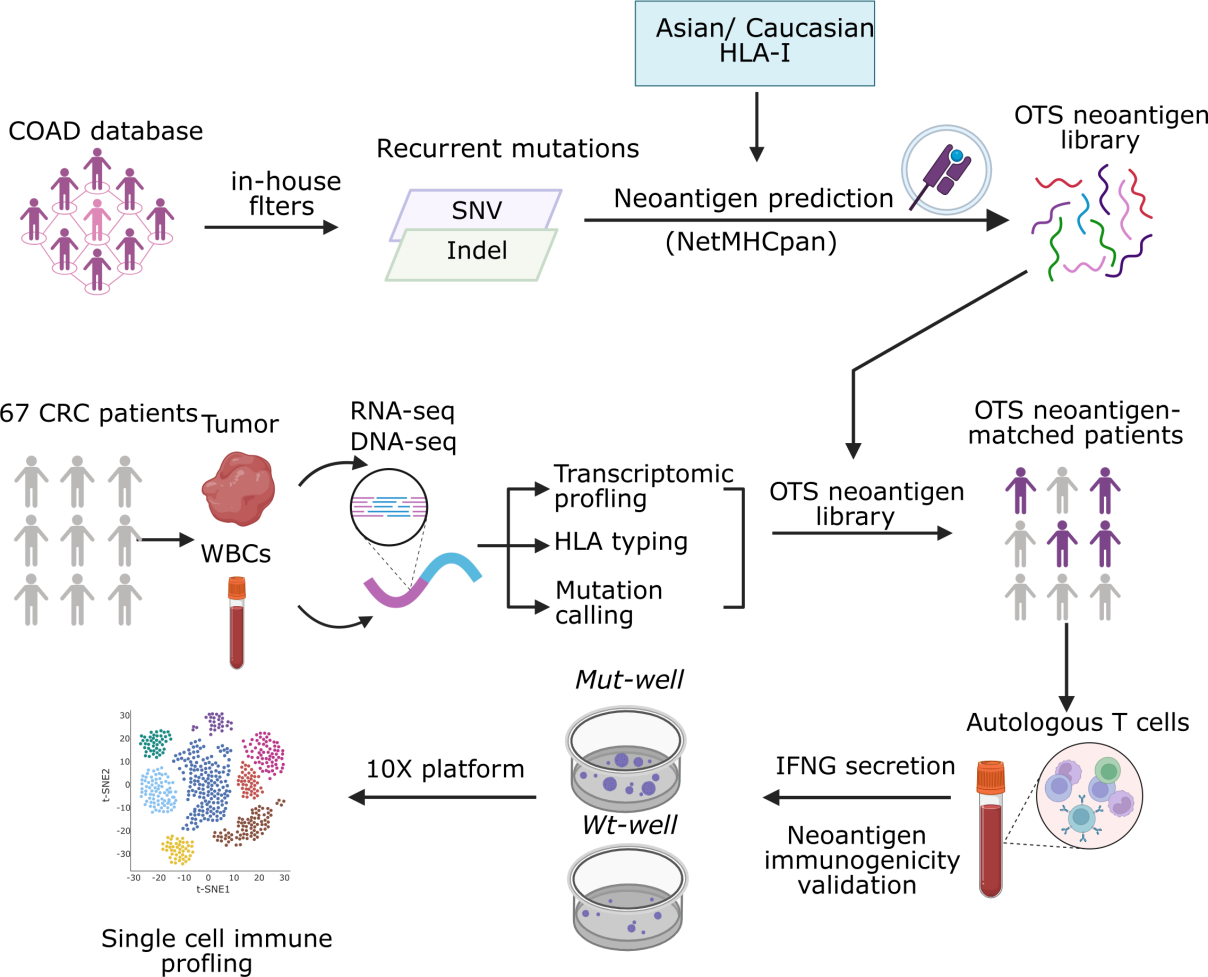
Schematic overview of the development and validation of an off-the-shelf (OTS) neoantigen panel for colorectal cancer (CRC). Recurrent mutations (SNVs and indels) were identified from CRC datasets using in-house filters and screened for predicted HLA-I binding (NetMHCpan) across Asian and Caucasian HLA alleles to generate an OTS neoantigen library. In a cohort of 67 CRC patients, tumor tissue and matched white blood cells (WBCs) underwent RNAseq and DNAseq for transcriptomic profiling, HLA typing, and mutation calling. Patients carrying both the mutation and the corresponding HLA allele from the OTS library were selected for immunogenicity testing. Autologous T cells were stimulated with mutant (Mut-well) or wild-type (Wt-well) peptides, and IFN-γ secretion was measured to assess neoantigen reactivity. Reactive T cells then underwent single-cell immune profiling (10x Genomics) to characterize TCR clonotypes and immune phenotypes.

To address the limitations of existing neoantigen panels, we developed a comprehensive strategy for shared neoantigen discovery that includes both high-frequency driver mutations and lower-frequency recurrent mutations with strong predicted binding affinities. In addition, unlike previous approaches that rely on a narrow set of HLA alleles, our method selects peptides predicted to bind across a diverse range of HLA-I alleles, including A, B, and C loci, better represent the genetic diversity of HLA class I alleles. We specifically selected HLA alleles that cover over 90% of individuals in East and Southeast Asia and Caucasian, enabling the development of an off-the-shelf neoantigen panel with broad clinical applicability. To validate this strategy, we assessed neoantigen coverage and T cell responses in a cohort of 67 CRC patients from a hospital in Vietnam, using single-cell RNA sequencing to analyze T cells after stimulation with the predicted peptides (**Figure 1**). This innovative and inclusive strategy uniquely integrates broad mutation coverage with population-specific HLA diversity, significantly enhancing the potential to elicit effective immune responses and paving the way for universally applicable neoantigen-based immunotherapies in colorectal cancer.

## Materials and methods

### Tumor mutation data

Somatic mutation data for colorectal cancer were obtained from The Cancer Genome Atlas (TCGA). Mutation Annotation Format (MAF) files for 308 colorectal adenocarcinoma (COAD) tumor samples were downloaded from https://xenabrowser.net/ and processed using annotations from the TCGA PanCancer Atlas project to ensure consistent genomic interpretation and standardized sample metadata (13).

### Mutation profile analysis

Mutated genes were classified as oncogenes or tumor suppressors based on COSMIC Cancer Gene Census (CGC) annotations; genes not in CGC were labeled “Unclassified” and excluded from functional analyses. Variant effects were predicted using Ensembl VEP (14).

To assess co-occurring and mutually exclusive mutation patterns in CRC, we performed pairwise co-mutation analysis using protein-coding somatic mutations from whole-exome sequencing data. Recurrent mutations were defined as non-synonymous variants (missense, nonsense, or frameshift) present in ≥2 tumor samples. For each mutation pair, a 2×2 contingency table was constructed to capture their co-occurrence across the cohort. Statistical significance was assessed using a two-sided Fisher’s exact test, with the strength and direction of association measured by the odds ratio (OR), where OR > 1 indicates co-occurrence and OR< 1 suggests mutual exclusivity. Multiple testing was corrected using the Benjamini-Hochberg method, and gene pairs with FDR-adjusted p-values< 0.05 were considered significant, following the framework described earlier (15). Significant pairs were visualized in a triangular heatmap: the upper triangle showed log-transformed ORs, and the lower triangle displayed corresponding FDR values.

To prioritize mutations with established relevance to cancer biology, we curated a reference annotation from the Cancer Gene Census (CGC) Hallmarks of Cancer database (https://cosmic-blog.sanger.ac.uk/hallmarks-cancer/). TCGA somatic mutations were aggregated at the gene–mutation–sample level to quantify recurrent events. Gene symbols were then matched to CGC entries, and each gene was assigned functional annotations corresponding to curated cancer hallmarks. Only genes with at least one CGC hallmark annotation were retained for downstream analyses, while genes lacking CGC hallmark annotations were excluded. This filtering step ensured that subsequent analyses focused exclusively on mutations affecting genes with established cancer relevance (**Supplementary Table S1**).

### HLA allele frequency analysis across Caucasian and Asian populations

To evaluate HLA allele distributions across ethnic groups, we retrieved population-specific frequency data from the IPD-IMGT/HLA Database (https://www.ebi.ac.uk/ipd/imgt/hla/), a curated source of high-resolution HLA genotyping. Populations were grouped as Caucasian (Belgium, Czech Republic, United States) and Asian (Hong Kong, China, Malaysia, Taiwan, Thailand, Vietnam). For each country, we collected allele frequencies for HLA-A, HLA-B, and HLA-C loci, giving priority to studies with larger sample sizes and higher-resolution HLA typing when multiple datasets were available.

### *In silico* prediction of HLA binding affinity

We predicted 8- to 11-mer epitopes binding to HLA-I (A, B, or C) using NetMHCpan-4.1. Neoantigen candidates were then prioritized based on their predicted binding affinity scores (measured in nM) for downstream analysis (16). The prioritization process involved calculating the percentile ranking of each neoantigen’s binding affinity score within the distribution of scores for the corresponding HLA allele. Neoantigen candidates with a percentile rank lower than 2% were considered as potential binders, while those with ranks below 0.5% were classified as strong binders (16).

To estimate the population-level frequency of each neoantigen, we included only HLA-I alleles predicted to bind with high affinity (percentile rank< 2%) using NetMHCpan-4.1. For each mutation-HLA pair meeting this threshold, we calculated the product of the mutation frequency and the corresponding HLA allele frequency in the target population. The overall neoantigen frequency was then computed as the average of these products across all predicted binding HLA-I alleles: $Neoantigen\;frequency_{population}=\frac{1}{n}\sum_{i=1}^{n}\left[Mutation\;Frequency\;\times \;HLA_{i}\;Frequency\right]$, where n is the number of predicted binding HLA-I alleles.

### Tumor biopsy and peripheral blood collection

A total of 67 CRC patients were enrolled at the University Medical Center in Ho Chi Minh City between June 2022 and May 2025. CRC diagnosis was confirmed by abnormal colonoscopy and histopathology, with staging based on American Joint Committee on Cancer and International Union for Cancer Control guidelines. All patients provided written informed consent for tumor and blood sample collection. Clinical data, including demographics, cancer stage, and pathology, were extracted from medical records (**Supplementary Table S2**). The study was approved by the Ethics Committee of the University of Medicine and Pharmacy at Ho Chi Minh City, with ethics approval number 316/HĐĐĐ-ĐHYD. Fresh tumor specimens were collected post-biopsy or resection and preserved in RNAlater (Thermo Fisher Scientific, Japan). For all patients, 10 mL of peripheral blood was collected before surgery in Heparin tubes.

### Targeted DNA and RNA sequencing

DNA and RNA were extracted using either the AllPrep DNA/RNA Mini Kit (Qiagen, Germany). Matched genomic DNA from white blood cells (WBCs) was isolated from buffy coat using the GeneJET Whole Blood Genomic DNA Purification Mini Kit (Thermo Fisher, MA, USA). Genomic DNA from paired tumor tissues and WBCs was used to construct DNA libraries with the ThruPLEX Tag-seq Kit (Takara Bio, USA), followed by hybridization with pre-designed probes targeting 95 CRC-related genes (Integrated DNA Technologies, USA), based on the COSMIC database. Libraries were pooled and sequenced on the DNBSEQ-G400 platform (MGI, Shenzhen, China) with 2×100 bp paired-end reads at an average coverage of 200× (range: 89–968×).

Total RNA was enriched for poly(A)+ RNA using the NEBNext Poly(A) mRNA Magnetic Isolation Module (New England Biolabs, MA, USA) and used to construct libraries with the NEBNext Ultra Directional RNA Library Prep Kit for Illumina. RNA libraries were sequenced on the MGI platform with 2×100 bp paired-end reads at a depth of 50×.

### Variant calling from DNA-seq and RNA-seq data

Somatic variants were identified using a pipeline based on published methods (17). For DNA-seq data, DRAGEN (Illumina) was used in tumor–normal mode with default filters. Common germline variants (from dbSNP, 1000 Genomes, and matched WBC DNA) and variants in immunoglobulin/HLA regions or synonymous mutations were excluded. Only mutations with ≥2% variant allele frequency (VAF) in fresh-frozen DNA were retained. For RNA-seq data, VarScan2 (18) was used in tumor–normal mode. Filters included PASS status, exclusion of population SNPs, minimum depth ≥10×, VAF ≥2%, and removal of synonymous or HLA-region variants. Reads were processed with Samtools (v1.10) and Picard (v2.25.6), and variants were manually reviewed in IGV (v2.8.2). Final VCFs from DRAGEN and VarScan2 were annotated using Ensembl VEP [v105 (14)].

DNA-seq variant calling was performed using DRAGEN for detection of single-nucleotide variants (SNVs) and insertions/deletions (INDELs) (17). RNA-seq–based variant calling was performed using VarScan, selected based on a previously published benchmarking study comparing VarScan and MuTect (17).

### Microsatellite instability analysis

MSI status was determined using fluorescent multiplex PCR with the MSI Analysis System v1.2 (Promega, USA), targeting five mononucleotide markers: BAT-25, BAT-26, MONO-27, NR-21, and NR-24. Genomic DNA was extracted from tumor tissues and matched WBC samples. PCR products were analyzed on a SeqStudio Genetic Analyzer (Thermo Fisher Scientific, USA). Samples with instability in ≥2 markers were classified as MSI-high (MSI-H), while those with one or no unstable markers were considered microsatellite stable (MSS).

### Isolation, culture, and stimulation of PBMCs with long peptides

PBMCs were isolated from 10 mL peripheral blood samples collected prior to surgery from seven patients using BD Vacutainer Heparin Tubes (BD Biosciences, NJ, USA). PBMCs were separated by density gradient centrifugation using Lymphoprep (STEMCELL Technologies), then resuspended in FBS/10% DMSO at 7–10 × 10^6^ cells/mL and cryopreserved in liquid nitrogen. Thawed PBMCs were cultured in AIM-V medium (Gibco, USA) supplemented with 10% FBS (Cytiva, USA) and DNase I (1 μg/mL, STEMCELL Technologies, Canada). 10^5^ PBMCs were plated in 96-well round-bottom plates with AIM-V medium supplemented with 10% FBS, 10 mM HEPES, and 55 μM β-mercaptoethanol and rested overnight. Cells were stimulated with GM-CSF (2000 IU/mL, Gibco, USA) and IL-4 (1000 IU/mL, Invitrogen, USA) for 24 hours, followed by LPS (100 ng/mL, Sigma-Aldrich, USA), IFN-γ (10 ng/mL, Gibco), and peptides (5 μM) for an additional 12 hours. On the next day, IL-7, IL-15, and IL-21 (10 ng/mL each, Peprotech, USA) were added. Peptides were restimulated in fresh media containing IL-7, IL-15, and IL-21 every three days, for a total of three stimulations. On day 12, cells were restimulated with peptides in cytokine-free media, and ELISpot assays were performed on day 13.

### Single-cell T-cell receptor and RNA sequencing

V(D)J TCR sequencing was performed to identify V, D, and J gene segments. Wells containing >100,000 cells from mutant and wildtype groups were selected. Dead cells were removed using the MojoSort Human Dead Cell Removal Kit (BioLegend, US). Cells were resuspended and adjusted to a concentration of ~700–1,200 cells/μL to optimize recovery and maintain >90% viability. Single-cell suspensions were mixed with RT Master Mix and loaded onto a Chromium Next GEM Chip A (10x Genomics, US) to generate gel bead-in-emulsion (GEM) droplets. Cell lysis and barcoded reverse transcription occurred within each GEM. Indexed cDNA was recovered and amplified by PCR. cDNA quality and concentration were assessed using the Quantus Fluorometer (Promega, US). Enriched TCR products were used for library construction and sequenced on an Illumina NovaSeq platform with 2 × 300 bp paired-end reads, generating ≥5,000 read pairs per cell. Library size was validated using the TapeStation system (Agilent, US). VDJ data were processed using the Cell Ranger pipeline.

Indexed cDNA from single-cell RNA sequencing was amplified by PCR, and 50 ng of cDNA was used to construct 5′ gene expression libraries. Samples were indexed and sequenced on an Illumina NovaSeq platform with 2 × 300 bp paired-end reads, generating a minimum of 30,000 read pairs per cell. Libraries were validated using the TapeStation system (DNA ScreenTape Analysis, Agilent). Single-cell RNA data were preprocessed with Cell Ranger, and low-quality cells or those lacking sufficient clonotype information were excluded from downstream analysis (19, 20).

### Statistical analysis

All statistical analyses were performed using R (version 4.3.2) unless otherwise specified. Fisher’s exact test was used to evaluate the association between microsatellite instability (MSI-H vs MSS) and the presence of panel-derived mutations, as well as to assess pairwise co-occurrence or mutual exclusivity between recurrent somatic mutations. P-values from co-mutation analyses were adjusted using the Benjamini–Hochberg method to control the false discovery rate (FDR), with FDR< 0.05 considered statistically significant.

## Results

### Establishment and characterization of a panel of recurrent mutations in CRC

**Figure 2:**
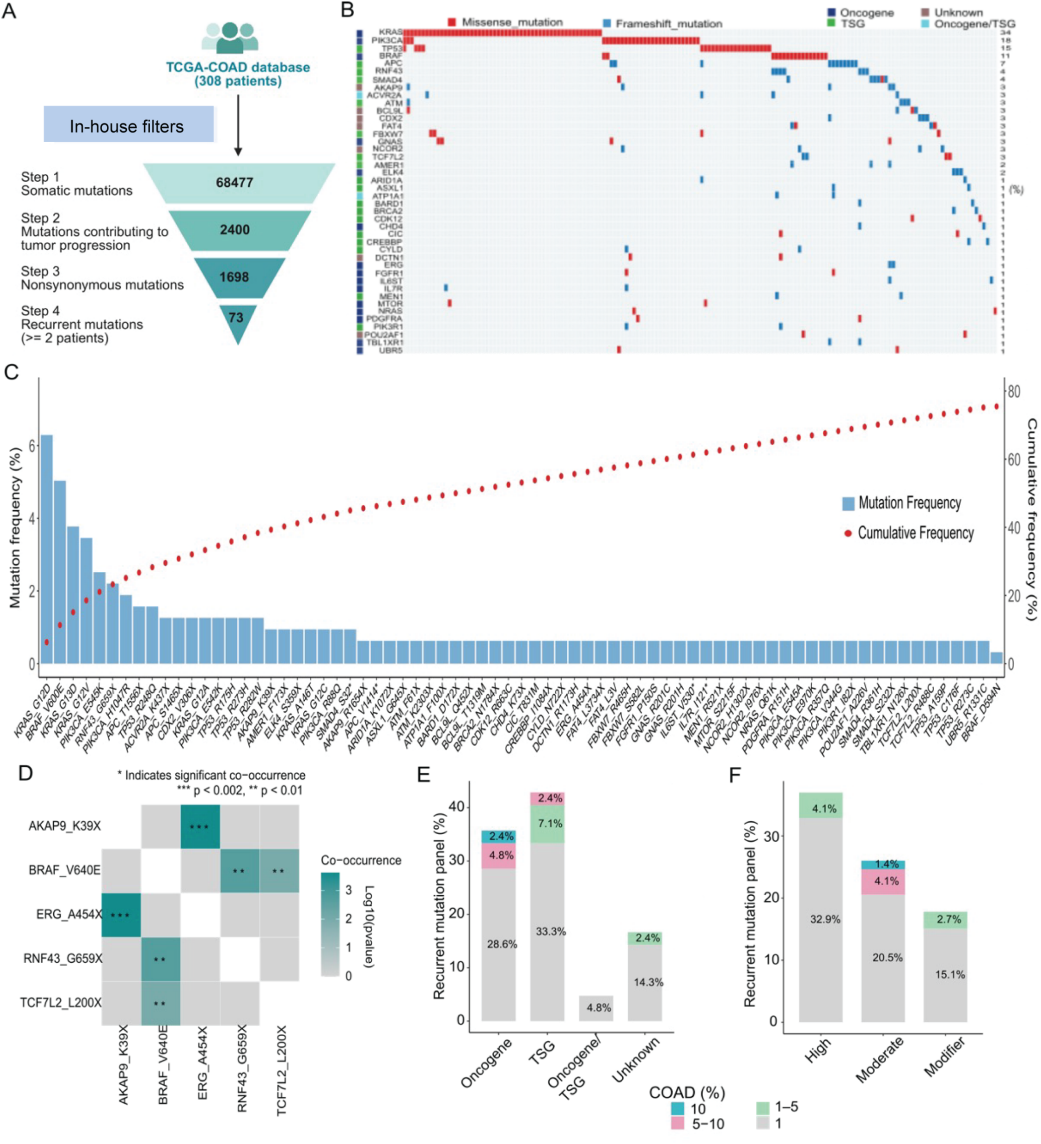
Characterization of a recurrent mutation panel from the TCGA-COAD dataset. **(A)** Workflow for identifying recurrent mutations in colorectal cancer using TCGA-COAD data (n = 308 patients). **(B)** Oncoplot of the 73 selected mutations across the TCGA-COAD cohort. Mutations are color-coded by type: missense (blue) and nonsense (red). Gene function is also color-coded: oncogenes (blue), tumor suppressor genes (TSGs, green), dual function genes (light blue), and genes of unknown function (brown). **(C)** Bar plot showing mutation frequency (blue bars) and cumulative patient coverage (red dots) for the 73 recurrent mutations. **(D)** Pairwise co-occurrence and mutual exclusivity matrix for frequently mutated genes in the panel. Statistically significant co-mutations are highlighted in green (**p < 0.01; ***p< 0.001). **(E)** Distribution of mutation recurrence by gene function category (oncogene, TSG, dual function, unknown), showing enrichment of panel mutations in cancer driving genes. Bar labels indicate the proportion within the panel; color segments represent proportions in the TCGA-COAD cohort. **(F)** Distribution of mutation recurrence by predicted functional impact (high, moderate, modifier), demonstrating enrichment of panel mutations with strong functional effects. Bar labels show proportions within the panel; color segments reflect TCGA-COAD cohort distribution.

To construct a recurrent mutation panel for CRC, we analyzed genomic data from 308 CRC patients in the TCGA-COAD dataset (**Figure 2A**). An initial pool of 68,477 somatic mutations was identified through DNA sequencing. Using an in-house filtering strategy that prioritized mutations within known cancer-related genes or those with predicted functional relevance, we refined the list to 2,400 candidate mutations potentially contributing to tumor progression. To further enrich for functionally significant variants, we retained only nonsynonymous mutations, which alter protein-coding sequences, thereby reducing the list to 1,698 mutations. Finally, we defined recurrent mutations as those detected in at least two patients, resulting in a curated panel of 73 recurrent mutations.

These mutations were mapped to 42 genes, which were classified into 18 tumor suppressor genes (TSGs), 15 oncogenes, 2 dual-function genes, and 7 genes of unknown function, based on annotations from COSMIC and relevant literature (21) (**Figure 2B**). Among these, *KRAS* (34% of patients), *PIK3CA* (18%), and *TP53* (15%) were identified as the most frequently mutated genes, primarily involving missense variants (**Figure 2B**). KRAS_G12D (6%) was the most common variant, followed by BRAF_V600E (5.2%), KRAS_G13D (3.9%), and KRAS_G12V (3.5%) (**Figure 2C**). The 73-mutation panel provides 78% coverage of the TCGA-COAD cohort, as indicated by the cumulative distribution curve in the plot (**Figure 2C**).

To assess co-occurrence patterns within the panel, we generated a triangular co-mutation plot (**Supplementary Figure 1A**). Genes were ranked by descending mutation frequency on both axes, and co-mutation strength was calculated using Fisher’s exact test, with log-odds ratios displayed in the lower triangle. We identified three significantly co-mutated pairs (p< 0.05): BRAF_V640E–RNF43_G659X (OR = 18.7, p = 0.0009), BRAF_V600E–TCF7L2_L200X (OR = ∞, p = 0.006), and ERG_A454X–AKAP9_K39X (OR = ∞, p = 0.0001) (**Figure 2D**). The BRAF_V600E- RNF43_G659X was previously established as part of the mutational profiles of MSI-high CRC (22), while the ERG_A454X–AKAP9_K39X and BRAF_V640E–TCF7L2_L200X co-mutation represents a potentially novel biological interaction.

We next assessed the functional impact of the mutations in the panel. The highly recurrent mutations (frequency > 5%) are enriched in oncogenes (**Figure 2E**) and exhibit moderate functional impact (**Figure 2F**). Collectively, using TCGA data, we established a panel of 73 mutations across 42 genes characterized by high recurrence and functional relevance in CRC patients, achieving a cumulative coverage of 78%.

### *In silico* prediction of neoantigen from recurrent mutations based on HLA-I alleles in Asian and Caucasian populations

**Figure 3:**
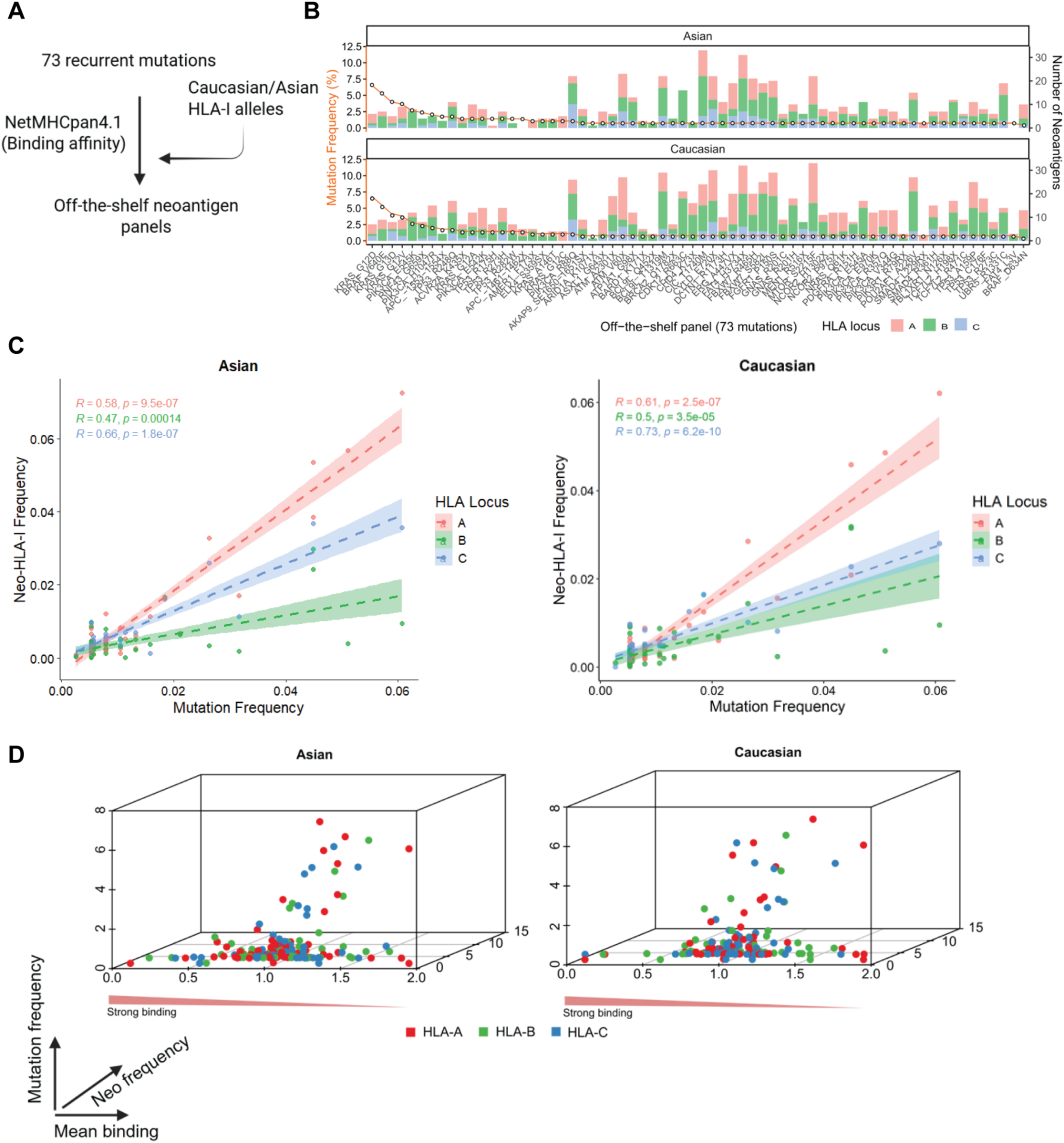
Mutation frequency and neoantigen presentation across HLA-I loci in Asian and Caucasian populations. **(A)** Workflow for establishing the off-the-shelf neoantigen panel. **(B)** Stacked bar plots showing the number of predicted HLA-I binders (neoantigens) per mutation for Asian (top) and Caucasian (bottom) populations. Mutations are ordered by descending mutation frequency. HLA loci are color-coded: A (red), B (green), and C (blue). The orange line indicates mutation frequency (%) across the panel. **(C)** Scatter plots showing the correlation between mutation frequency and cumulative predicted HLA-I neoantigen frequency in Asian (left) and Caucasian (right) cohorts. Neoantigen frequency increases proportionally with mutation frequency across all HLA loci, as assessed by Spearman’s rank correlation. Regression lines with confidence intervals are shown for each locus. **(D)** 3D scatter plots showing the relationship among mutation frequency (x-axis), neoantigen-HLA binding rank (y-axis), and HLA-I locus frequency (z-axis, color-coded by locus). These plots visualize the distribution and locus-specific density of predicted neoantigen presentation across populations, highlighting the dominance of HLA-A–restricted neoantigens in both ethnic groups.

To evaluate the population-wide neoantigen presentation potential of our 73 recurrent mutations, we employed NetMHCpan 4.1 to predict peptide–HLA class I binding affinities (**Figure 3A**). Binding predictions were performed using commonly expressed HLA-I alleles in Caucasian and Asian populations, selected to represent approximately 90% of each group (**Supplementary Figure 1B**). Notably, all 73 mutations generated at least one predicted neoantigen in both population groups. The number of HLA-restricted neoantigens per mutation varied substantially, ranging from 1 to 33, with HLA-A and HLA-B alleles contributing the largest proportion in both Asian and Caucasian populations (**Figure 3B**). Interestingly, the most frequent mutations—such as KRAS G12D (6–7 neoantigens), BRAF V600E (7–9 neoantigens), KRAS G13D (2–4 neoantigens), and KRAS G12V (9 neoantigens)—consistently produced fewer neoantigen candidates compared to several lower-frequency mutations (**Figure 3B**). This underscores the importance of evaluating both mutation prevalence and neoantigen yield when selecting candidates for a shared neoantigen panel.

We next analyzed the correlation between mutation frequency and neo-HLA-I frequency in both Caucasian and Asian populations (**Figure 3C**). In both cohorts, strong positive correlations were observed across all three HLA-I loci, with HLA-C showing the strongest correlation (R = 0.73, p = 6.2 × 10^−10^ for Caucasians; R = 0.66, p = 1.8 × 10^−7^ for Asians; **Figure 3C**). Despite HLA-C exhibiting the highest correlation, neoantigen frequency was consistently greater for HLA-A in both populations, suggesting that HLA-A alleles contribute more substantially to the overall neoantigen load, regardless of ethnicity (**Figure 3C**, **Supplementary Figure 1**).

To further assess the qualitative nature of these neoantigens, we evaluated the distribution of predicted binding affinity (expressed as percentile rank) in relation to both neoantigen and mutation frequencies (**Figure 3D**). We found that strong-binding neoantigens (percentile rank< 0.5%) were not predominantly associated with high-frequency neoantigens or high-frequency mutations. In both populations, mutations with the highest predicted binding strength tended to occur at lower frequencies, indicating a potential trade-off between mutation prevalence and immunogenic potential (**Figure 3D**). This observation does not contradict the off-the-shelf neoantigen concept but rather reflects a known biological constraint: many highly recurrent driver mutations generate peptides with suboptimal HLA binding or presentation (23). Therefore, to balance population coverage and immunogenic potential, we extended our shared neoantigen panel to include candidates with reasonable recurrence (≥1%) and strong predicted strong HLA binding (percentile rank<0.5%). While NetMHCpan-4.1 is a widely used tool for predicting peptide–HLA class I binding, it primarily evaluates binding affinity and does not directly model T-cell receptor (TCR) recognition, which is a key determinant of immunogenicity. To further assess the immunogenic potential of our neoantigen panel, we extended our analysis using additional prediction frameworks, including PRIME2.0 (24), and DeepImmuno (25), which incorporate peptide features beyond HLA binding and are designed to capture aspects relevant to TCR recognition and ACME (26), which predicts peptide–MHC class I binding and neoantigen presentation. For each tool, neoepitopes were considered positive when the mean prediction score of the mutant peptide exceeded that of the corresponding wild-type peptide. Under this criterion, all NetMHCpan-filtered neoepitopes were consistently supported by ACME and PRIME2.0 (73/73 (100%) concordance), while DeepImmuno supported (69/73 – 94.5%) of the filtered neoepitopes (**Supplementary Figure 2**). The high concordance across independent immunogenicity prediction frameworks increases the likelihood that the selected neoantigen candidates are capable of eliciting patient T-cell responses.

### Coverage of the off-the-shelf neoantigen panel in Vietnamese CRC patients

**Figure 4:**
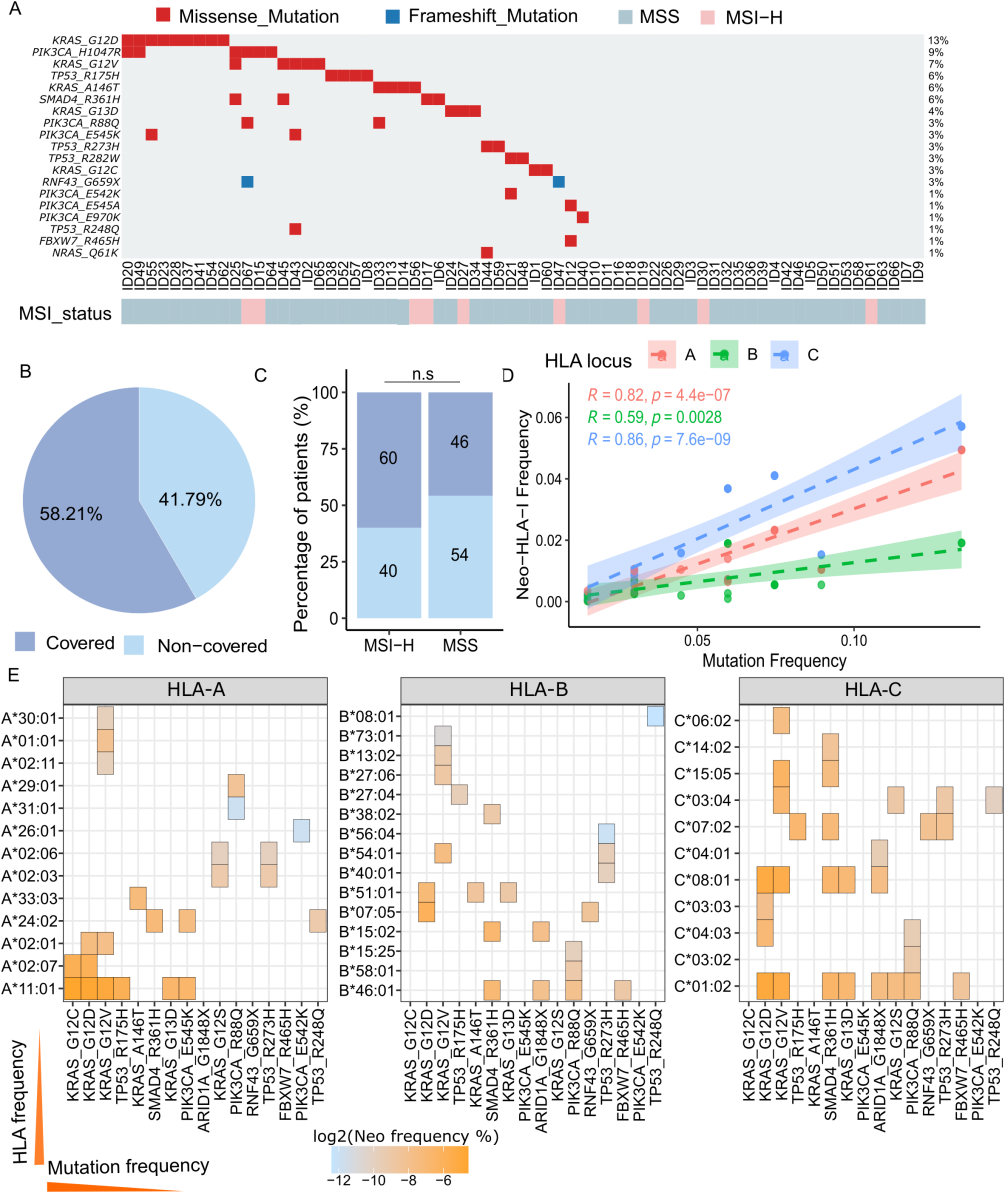
Validation of the off-the-shelf neoantigen panel in a Vietnamese CRC cohort (n = 67). **(A)** Oncoplot showing the distribution of panel mutations among 67 Vietnamese CRC patients. Each column represents a patient, each row a mutation. Mutations are color-coded by type: missense (red) and nonsense (blue). Bottom annotation indicates microsatellite stability (MSS) status. **(B)** Pie chart showing the proportion of patients with at least one matched OTS neoantigen. **(C)** Bar chart comparing the number of patients with at least one panel mutation between MSS and MSI subgroups. p > 0.5 (Fisher’s test, not significant). **(D)** Correlation between mutation frequency and predicted HLA-I neoantigen frequency across the Vietnamese cohort. Linear regression was performed separately for HLA-A, -B, and -C loci, as assessed by Spearman’s rank correlation. (R and p-values shown). **(E)** Heatmaps displaying the density and distribution of neoantigen-HLA binding predictions across HLA-A, -B, and -C alleles. Color gradient reflects the frequency of neoantigen-HLA interactions, with higher frequencies in orange.

To evaluate the applicability of our recurrent neoantigen panel in an independent population, we analyzed a cohort of 67 colorectal cancer (CRC) patients recruited in Vietnam for the presence of panel-included mutations. Coverage was defined at the patient level by the co-occurrence of a somatic mutation and at least one patient-specific HLA class I allele predicted to bind the corresponding neoepitope. A patient was considered “covered” only if they harbored at least one such mutation–HLA pair, rather than being counted based on mutation presence alone (**Supplementary Figure 3**). Using this definition, 58.21% of patients in the Vietnamese CRC cohort were predicted to have at least one mutation–HLA pair capable of producing a bindable neoepitope (**Supplementary Figure 3**, **Figures 4A, B**). The most frequently observed mutations were KRAS_G12D (13%), KRAS_G12V (7%), and TP53_R175H (6%) (**Figure 4A**), closely mirroring the mutation frequency distribution observed in the TCGA cohort (**Figure 2B**). The mutation landscape in this Vietnamese cohort was predominantly composed of missense mutations, with frameshift mutations limited to ARID1A_G1848X and RNF43_G659X (**Figure 4A**). We observed a trend toward differing prevalence of shared neoantigen panel between MSI-H and MSS tumors, however this does not reach statistical significance (p = 0.5; **Figure 4C**), suggesting that these neoantigens are not restricted to tumors with mismatch repair deficiency and may be broadly applicable across molecular subtypes.

We further assessed the correlation between neoantigen frequency and HLA or mutation frequency within this cohort. Consistent with previous observations, neoantigen frequency increased with mutation frequency across all three HLA loci (HLA-A: R = 0.97, p = 1.6 × 10^−14^; HLA-B: R = 0.69, p = 3.0 × 10^−4^; HLA-C: R = 0.90, p = 8.3 × 10^−1^; **Figure 4D**). These results confirm the reproducibility of our panel’s immunogenic features in an ethnically distinct cohort and support its potential utility for vaccine development across diverse populations. Notably, highly recurrent neoantigens were frequently presented by prevalent HLA-A and HLA-C alleles but were rarely associated with even the most common HLA-B alleles (**Figure 4D**). Specifically, neoantigen–HLA pairs involving KRAS mutations (KRAS_G12C and KRAS_G12D) were frequently associated with HLA-A*11:01, HLA-C*01:02, and HLA-C*08:01 (**Figure 4E**). The high recurrence of these neoantigen–HLA combinations suggests that certain alleles may serve as immunodominant presenters for common mutations, making them promising targets for population-scale vaccine strategies.

### Immunogenicity of panel-selected neoantigens in CRC patients

**Figure 5:**
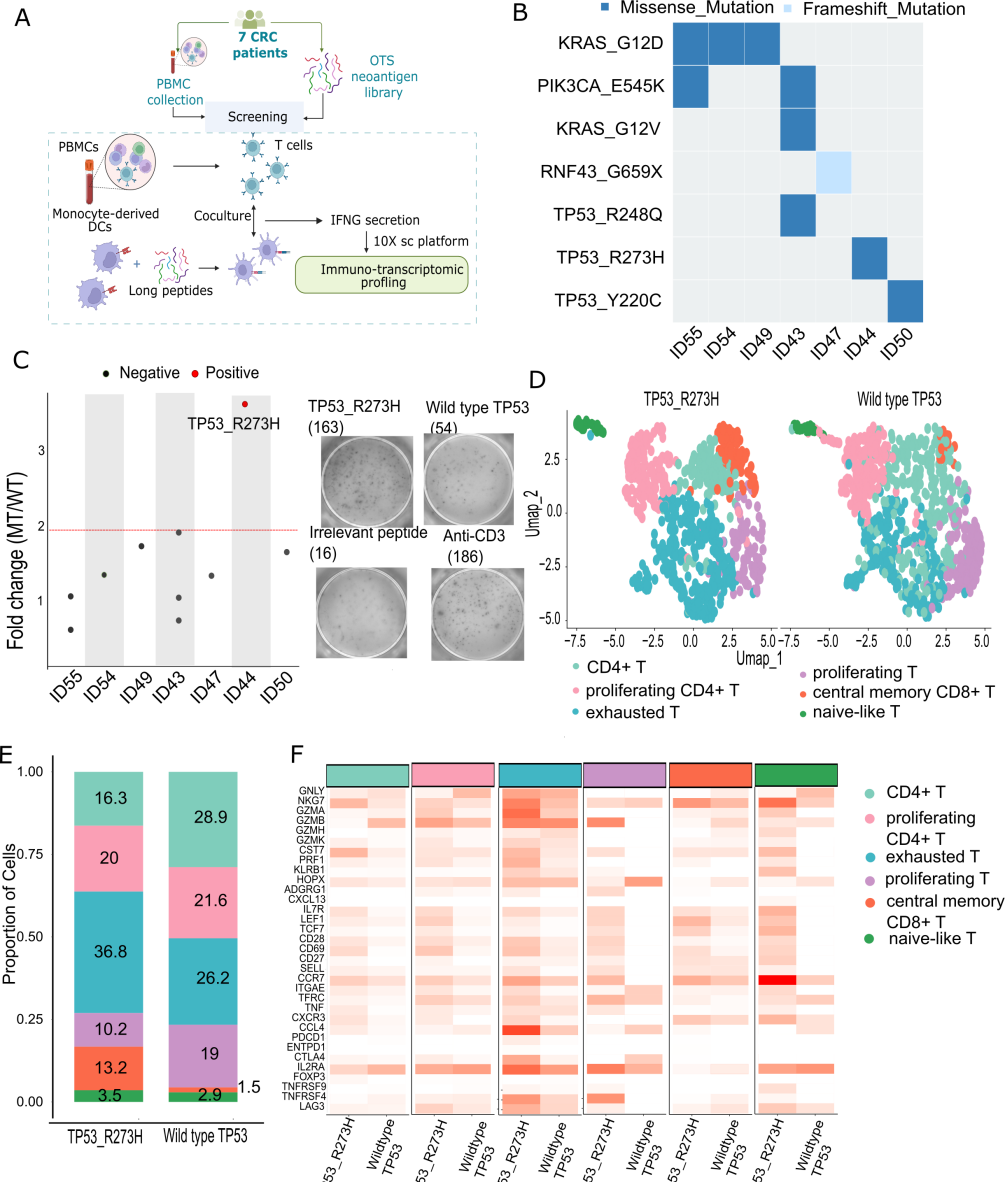
Functional validation and single-cell immune profiling of neoantigen-specific T cell responses in 7 CRC patients. **(A)** Schematic of the neoantigen screening workflow. **(B)** Oncoplot showing the distribution of panel mutations among the 7 Vietnamese CRC patients selected for immunogenicity testing. **(C)** ELISpot IFN-γ assay results showing fold change in spot counts per 1×10^5^ cells between mutant peptide stimulation and corresponding wild-type peptide. The red dashed line indicates a fold change threshold of 2, representing the cutoff for a positive immune response. Representative well images show spot formation indicating T cell reactivity. **(D)** UMAP projections of single-cell transcriptomic data from neoantigen-reactive T cells, showing distinct cluster distributions under TP53_R273H versus wild-type TP53 stimulation. **(E)** Cluster distribution of T cell subsets following TP53_R273H (left) and wild-type peptide stimulation (right). **(F)** Heatmap of differentially expressed genes across T cell clusters identified by single-cell RNA sequencing.

To experimentally validate the immunogenicity of selected recurrent neoantigens, PBMCs from CRC patients were stimulated *in vitro* with a neoantigen peptide library derived from our 73-mutation panel, followed by single-cell transcriptomic profiling to assess T cell activation (**Figure 5A**). In this study, we employed a PBMC-based immunogenicity assay in which monocytes were differentiated into immature dendritic cells using GM-CSF and IL-4, followed by maturation with LPS and IFN-γ to induce the expression of costimulatory molecules (CD80 and CD86) and antigen-presentation machinery required for effective priming of antigen-specific T cells. This approach is widely used in neoantigen vaccine and T-cell immunogenicity studies to provide a controlled and standardized antigen-presenting context (5, 27). Among CRC patients harboring mutations included in the panel, seven were selected based on the availability of sufficient PBMCs for testing. Of these, two patients (ID43 and ID55) carried more than one mutation from the panel, and one harbored a frameshift mutation (**Figure 5B**). Twenty-five–amino-acid-long peptides were used to stimulate PBMCs. While long peptides are preferentially presented via MHC class II molecules and thus primarily activate CD4^+^ T cells, they can be processed by antigen-presenting cells into shorter fragments that generate MHC class I-restricted epitopes, enabling cross-presentation mediated activation of CD8^+^ T cells (28, 29). Consistent with this, although CD4^+^ T-cell responses predominated in both the functional assays and single-cell analyses, CD8^+^ T cells contributing to IFN-γ induction were still detectable (**Supplementary Figures 6A, B**). Further stratification analysis of IFN-γ–producing CD8^+^ T-cell subsets suggested that these responses originated predominantly from early activated (proliferating), exhausted, and central memory compartments (**Supplementary Figures 6C, D**). All seven patients were evaluated for immune reactivity against neoantigen peptides, and T cell responses were assessed using IFN-γ ELISpot assays. A mutant-to-wild-type fold change greater than 2 was considered indicative of specific reactivity (**Figure 5C**). KRAS_G12D neoantigens were predicted in 3 of 7 patients, but none elicited an immunogenic response (**Figure 5C**). By contrast, only patient ID44 exhibited a positive immune response, specifically against TP53_R273H (**Figure 5C**). As shown in **Supplementary Figures 4**, **5**, TP53_R273H demonstrated strong HLA-I binding, high tumor VAF, and adequate gene expression - three parameters that collectively support its immunogenicity. This finding emphasizes the importance of integrating multiple features to accurately identify neoantigens with high immunogenicity that are capable of inducing strong T cell responses.

To gain deeper insight into the functional state of TP53_R273H-reactive T cells, cells from ELISpot-positive wells were isolated and analyzed by single-cell TCR RNA sequencing. UMAP projection plots (**Figure 5D**) identified six distinct T cell clusters shared between mutant and wild-type stimulation conditions: CD4^+^ T cells, proliferating CD4^+^ T cells, naïve-like T cells, exhausted T cells, proliferating T cells, and central memory CD8^+^ T cells. The mutant-stimulated group exhibited significant enrichment in both the exhausted T cell cluster and the central memory CD8^+^ T cell cluster, indicating functional polarization in response to antigen stimulation (**Figure 5E**). Single-cell transcriptomic analysis revealed the expansion of multiple T-cell subsets following neoantigen stimulation, with increased representation of both CD8^+^ and CD4^+^ populations and a dominant *IFNG* expression signal in mutant peptide–stimulated conditions compared with wild-type controls (**Supplementary Figures 6A, B**). Although *IFNG* expression was predominantly detected in CD4^+^ T cells, robust upregulation was also observed in CD8^+^ T cells, with significantly higher expression in response to the mutant peptide compared with the wild-type control, accompanied by induction of cytotoxic effector genes—consistent with MHC class I–restricted antigen recognition (**Supplementary Figures 6A, B**). These findings demonstrate that the ELISPOT-detected immune responses are mediated, at least in part, by functionally active CD8^+^ T cells, supporting their potential relevance for CD8^+^ T-cell–based cancer immunotherapy. Upon stimulation with mutant peptides, CD8^+^ T cells were reduced in the exhausted cluster (9.8% vs. 16%) but enriched in the proliferating cluster (9.6% vs. 2.4%) (**Supplementary Figure 7**). These findings suggest that CD8^+^ T cells within the proliferating compartment may contribute, at least in part, to the enhanced IFN-γ response observed following neoantigen stimulation (30). Differential gene expression analysis (**Figure 5F**) further revealed upregulation of key immune markers. Within the central memory CD8^+^ T cell cluster, elevated expression of *IL7R*, *LEF1*, *TCF7*, *CD69*, *CCR7*, and *TFRC* suggested stem-like memory potential. In the exhausted T cell cluster, high expression of cytotoxic effector genes, including *NKG7*, *GZMB*, *GZMA*, and *PRF1*, reflected an activated yet functionally exhausted phenotype. Our data show that panel-selected reactive neoantigen candidates can induce transcriptomic activation in patient-derived T cells, underscoring their potential relevance for cancer vaccine development.

## Discussion

Shared neoantigens represent a promising avenue for rapid and scalable immunotherapy in CRC. Unlike personalized neoantigen approaches, which require extensive patient-specific sequencing and custom vaccine development, shared neoantigens are derived from recurrent mutations across patients, enabling the creation of off-the-shelf vaccines (9, 31). This strategy offers significant advantages in terms of production efficiency, cost-effectiveness, and broader clinical applicability across larger patient populations. However, the development of shared neoantigen-based therapies is hindered by two major challenges: the scarcity of highly recurrent neoantigen candidates and the dependence of immunogenicity on both HLA allele distribution and TCR repertoire diversity (32, 33). To address these barriers, we curated a panel of highly recurrent neoantigens in CRC by integrating public genomic datasets with established neoantigen prediction algorithms. The panel was optimized for broad coverage by prioritizing mutations presented by common HLA class I alleles in both Asian and Caucasian populations. In line with prior studies (4, 34) the selected mutations were enriched for single nucleotide variants (SNVs) predicted to have moderate to high functional impact, particularly in oncogenes and tumor suppressor genes (TSGs). The recurrence of these mutations likely reflects their essential roles in tumorigenesis and positions them as attractive targets for immune-mediated tumor rejection. These mutations are also under strong selective pressure, contributing to their prevalence and potential therapeutic relevance. Finally, our cumulative coverage analysis (**Figure 2**) suggests that the panel has reached a saturation point, where inclusion of additional low-frequency mutations yields diminishing returns in terms of population-level HLA-I coverage. Moreover, we found that while the BRAF_V600E–RNF43_G659X co-mutation has been previously recognized as a characteristic feature of the mutational landscape in MSI-high CRC (22), the ERG_A454X–AKAP9_K39X and BRAF_V600E–TCF7L2_L200X co-mutations may represent potentially novel biological interactions that warrant further investigation. The findings from this study are particularly relevant for the development of neoantigen-based cancer vaccines, which represent a more scalable and cost-effective therapeutic strategy compared with personalized cell therapies.

Although highly recurrent mutations such as KRAS G12D, G13D, and BRAF V600E are appealing targets for immunotherapy due to their prevalence in CRC (**Figure 3**), our analysis highlights a key limitation: these mutations often generate few high-quality HLA-I restricted neoepitopes. This is largely attributed to unfavorable local sequence contexts surrounding the mutation sites, which hinder the generation of peptides with strong MHC-I binding affinity and efficient cell surface presentation. Consistent with this, Claeys et al. (23) showed that frequent driver mutations—including KRAS G12C/D/V, PIK3CA E545K, TP53 R175H, and BRAF V600E—tend to produce peptides with inherently weak HLA-I binding due to suboptimal anchor residues within their native sequences. These biochemical constraints compromise both the immunogenicity and the breadth of HLA coverage for such mutations, limiting their recognition by CD8^+^ T cells across diverse patient populations. This is supported by our immunogenicity testing (**Figure 5**), where only one of seven CRC patients exhibited a T cell response—and it was directed against TP53 R273H rather than KRAS G12D, despite the latter being the most common mutation in our panel. In this study, although the overall coverage of mutation-HLA pairs reached 58%, only a subset of neoantigen candidates elicited detectable immunogenic responses. The ability of a predicted neoantigen to become immunogenic likely depends on multiple factors, including (1) limited or absent cognate TCR repertoires in the tested PBMCs (35), (2) suboptimal antigen processing or presentation despite favorable binding predictions (35, 36), (3) lower clonality or antigen abundance (37), and (4) immune dysfunction or suppressive features in patient samples (38). Consistent with these principles, TP53_R273H in patient ID44 exhibited relatively higher gene expression and tumor VAF, generated a greater number of predicted neopeptides capable of binding to the patient’s HLA alleles, and yielded multiple positive immunogenicity predictions across three independent tools - DeepImmuno and PRIME, which explicitly model T-cell immunogenicity and TCR recognition potential, and ACME, which predicts peptide–MHC class I binding and neoantigen presentation (24–26). This convergence of favorable features likely explains why TP53_R273H elicited a detectable T-cell response while other recurrent mutations did not and underscores the importance of multi-parameter neoantigen prioritization beyond mutation–HLA coverage alone (**Supplementary Figure 4**, **Figure 5C**). Our findings reinforce that recurrence alone does not determine neoantigen immunogenicity. Although KRAS_G12D is a highly recurrent oncogenic driver, it failed to elicit detectable T-cell responses in our validation cohort. Comparative analysis with the immunogenic TP53_R273H neoantigen revealed that KRAS_G12D had lower antigen abundance - reflected by reduced tumor gene expression and variant allele frequency (**Supplementary Figure 4**), and lacked consistent immunogenicity predictions across multiple tools (**Supplementary Figure 5**). These observations are consistent with clinical data from shared neoantigen vaccine studies, where TP53-derived neoantigens preferentially induced T-cell responses over KRAS neoantigens (39). Together, these results highlight a hierarchy of immunodominance in which neoantigen quality, rather than mutation frequency, governs effective T-cell recognition, emphasizing the importance of multi-dimensional neoantigen evaluation for immunotherapy target selection. We also found similar patterns have been observed in early phase KRAS neoantigen vaccine trials, where T cell responses were often restricted to patients with specific HLA types, such as HLA-A11:01 or HLA-C08:02 (40–43). Together, these findings suggest that mutation frequency alone is insufficient to predict neoantigen potential. In fact, our data indicate that several lower frequency mutations generate a greater number of high affinity, HLA-I restricted epitopes, thus offering broader population coverage. This highlights the importance of considering both mutation prevalence and immunogenic fitness when designing off-the-shelf neoantigen based therapies.

We prospectively evaluated the utility of our neoantigen panel in a cohort of 67 Vietnamese CRC patients, achieving a 58% coverage rate (**Figure 4**). Although slightly lower than the 78% coverage observed in the TCGA-COAD dataset, this still represents substantial representation in an independent and ethnically distinct population. The consistent coverage across both Western and Asian cohorts highlights the panel’s broad applicability and supports its potential for real world clinical deployment in genetically diverse settings (**Figure 4**). Compared to previous efforts, such as the pan-cancer off-the-shelf panel proposed by Mauriello et al. (44), which provides coverage ranging from 4% to 50% depending on cancer type, our CRC focused panel offers improved translational relevance. It achieves high HLA-I population coverage (~90%) by incorporating a comprehensive set of 18 HLA-A, 34 HLA-B, and 16 HLA-C alleles spanning both Caucasian and Asian populations. While prior studies primarily relied on *in silico* predictions, our work includes functional validation using primary T cells from CRC patients, demonstrating antigen specific responses to selected neoantigens. Transcriptomic profiling of reactive T cells revealed a gene expression signature associated with anti-tumor activity, including elevated levels of cytotoxic and effector related markers (**Figure 5**). Notably, this response included both central memory CD8^+^ T cells and an exhausted like subset, suggesting that optimal cancer vaccines should aim to prime both long-lived memory cells and short term cytotoxic effectors (45–47). Our single-cell RNA-seq analysis shows that the exhausted (Tex) and proliferating (Tprolif) T-cell states are not restricted to a single lineage, but instead comprise both CD4^+^ and CD8^+^ T cells. Stratified analysis based on CD4 and CD8 expression revealed that CD4^+^ T cells account for the majority of cells within both Tex and Tprolif clusters (**Supplementary Figure 7**), providing a mechanistic explanation for the substantial contribution of CD4^+^ T cells to the IFN-γ signal detected by ELISPOT assays. The induction of those populations following stimulation with TP53_R273H further supports its candidacy as a clinically relevant shared neoantigen capable of eliciting durable and therapeutically potent T cell responses.

Our study has several limitations. First, we employed the conventional NetMHCpan tool for neoantigen prediction, which primarily estimates HLA-peptide binding affinity without incorporating TCR recognition or tumor micro environment (TME) influences. As a result, our assessment of immunogenicity may be incomplete. Future efforts should integrate predictive models of TCR binding and account for TME factors such as immune suppression and stromal modulation to enhance the accuracy of neoantigen selection. Second, the limited sample size for immunogenicity validation (n = 7) restricts the generalizability of our findings. This constraint arose from limited availability of viable T cells from many patients, which precluded a comprehensive assessment of immune responses across the entire cohort. Larger prospective cohorts will be essential to validate T cell responses at scale and further refine the neoantigen panel. Third, we acknowledge that repeated peptide stimulation of PBMCs during prolonged *in vitro* culture can preferentially expand rare antigen-reactive T cells and therefore may not fully reflect the composition or frequency of the pre-existing *in vivo* T-cell repertoire. To address this limitation, our analyses emphasized relative comparisons between mutant and corresponding wild-type peptides assessed under identical culture conditions, rather than relying on absolute measures of response magnitude. In addition, negative control cultures without peptide stimulation were included throughout the extended culture period to monitor nonspecific or bystander T-cell activation. Importantly, the differential responses observed between mutant and wild-type peptides (**Figure 5C**) were supported by concordant predictions across multiple independent immunogenicity frameworks, lending confidence to the robustness of these relative comparisons. Nonetheless, prolonged *in vitro* stimulation cannot fully recapitulate the complexity of *in vivo* antigen presentation and immune regulation. Future studies incorporating earlier time-point readouts, ex vivo functional assays, or *in vivo* validation models will be important to further establish the physiological relevance of the identified neoantigen-specific T-cell responses. In conclusion, by addressing challenges related to candidate scarcity and immunogenic variability across HLA class I types, and by incorporating functional validation in patient-derived T cells, our study supports the concept of public neoantigens in colorectal cancer while emphasizing the need for integrated computational and experimental approaches to identify candidates most likely to elicit functional T-cell responses for off-the-shelf cancer vaccine development.

## Data Availability

The datasets presented in this study can be found in online repositories. The names of the repository/repositories and accession number(s) can be found below: BioProject via accession ID PRJNA1005034.

## Supplementary material

Supplementary Table 1 List of genes functionally associated with hallmarks of cancer.

Supplementary Table 2 Demographic and clinical characteristics of 67 patients with CRC.

Supplementary Figure 1 Co-mutation landscape and HLA-I allele coverage for the recurrent neoantigen panel. **(A)** Pairwise co-occurrence and mutual exclusivity matrix for frequently mutated genes in the panel. Statistically significant co-mutations are highlighted in green (***p< 0.001). **(B)** Top panel: Cumulative frequency of HLA-A, -B, and -C alleles among Asian populations, covering up to 90% cumulative allele frequency. Alleles are color-coded by whether they are shared with (blue) or unique compared to (pink) the Caucasian population. Bottom panel: Same as above, shown for comparison across allele categories and loci.

Supplementary Figure 2 Concordance of immunogenicity predictions for neoantigen candidates in the off-the-shelf (OTS) panel across four prediction tools (NetMHCpan-4.1, ACME, PRIME2.0, and DeepImmuno). Neoepitopes were considered positive when the predicted score of the mutant peptide exceeded that of the corresponding wild-type peptide.

Supplementary Figure 3 HLA class I binding landscape of neoantigen candidates in the off-the-shelf (OTS) panel across the 67 patients in the cohort study. Heatmap showing predicted peptide-HLA class I binding across HLA-A, HLA-B, and HLA-C loci for neoantigen candidates included in the OTS panel across 67 CRC patients. Columns represent individual patients, and rows represent HLA class I loci. Purple squares indicate positive predicted peptide-HLA binding, while grey squares indicate no predicted binding under the applied threshold.

Supplementary Figure 4 Expression level, tumor VAF, and predicted peptide–HLA binding affinity of recurrent mutations in 7 patients included in immunogenicity validation assays. Each point denotes a short peptide derived from the corresponding synthetic long peptide used for *in vitro* T-cell stimulation.

Supplementary Figure 5 Immunogenicity predictions for neoantigens derived from mutations detected in seven patients included in the immunogenicity validation assays. For each prediction tool (DeepImmuno, ACME, and PRIME 2.0), colored squares indicate positive immunogenicity predictions for patient-specific HLA–neoantigen pairs, whereas grey squares indicate negative predictions. A positive prediction was defined as a statistically significant increase (p< 0.05) in immunogenicity scores for mutant-derived short peptide–HLA pairs compared with the corresponding wild-type peptides.

Supplementary Figure 6 Single-cell characterization of neoantigen-reactive T cells following mutant and wild-type peptide stimulation. **(A)** UMAP visualization of single-cell RNA sequencing data showing CD4^+^ and CD8^+^ T-cell populations after stimulation with mutant (left) or corresponding wild-type (right) peptides. Cells are colored by T-cell subset (CD4^+^, teal; CD8^+^, orange). **(B)** Comparison of IFNG expression levels in CD4^+^ and CD8^+^ T cells following stimulation with mutant versus wild-type peptides. Box plots show higher IFNG expression in mutant peptide stimulated conditions for both CD4^+^ and CD8^+^ T cells, with statistically significant differences indicated (****P< 0.0001; ***P< 0.001). **(C)** UMAP visualization of single-cell transcriptomes highlighting CD8^+^ T-cell subsets, including exhausted (CD8^+^ Tex), central memory–like (CD8^+^ Tcm), and proliferating (CD8^+^ Tprolif) populations. Cells are shown separately for mutant and wild-type conditions, with non-CD8 T cells labeled as “Other.” **(D)** Quantification of IFNG expression levels in CD8^+^ Tex, CD8^+^ Tcm, and CD8^+^ Tprolif subsets comparing mutant and wild-type conditions. Statistical significance was assessed using a two-sided Wilcoxon rank-sum test; *P< 0.05; UD: Undetermined.

Supplementary Figure 7 Single-cell profiling of neoantigen-reactive T cells following mutant versus wild-type peptide stimulation. **(A)** UMAP representation of single-cell RNA sequencing data showing T-cell clusters following stimulation with mutant (left) or corresponding wildtype (right) peptides. Distinct T-cell subsets are highlighted, with circled regions indicating exhausted T (Tex) and proliferating T cells (Tprolif) **(B)** Proportional composition of CD4^+^ and CD8^+^ T cells among exhausted T cells (Tex) following mutant and wild-type peptide stimulation. Percentages indicate the relative fraction of each subset. **(C)** Proportional composition of CD4^+^ and CD8^+^ T cells among proliferating T cells (Tprolif) under mutant and wild-type peptide–stimulated conditions.
