# Supplementary figures and images for: Identification of public neoantigens with broad HLA class I coverage as candidates for off-the-shelf cancer vaccine development in colorectal cancer
Source: Front Immunol. 2026 Feb 18;17:1686224. doi: 10.3389/fimmu.2026.1686224 (PMC12957132; doi:10.3389/fimmu.2026.1686224)

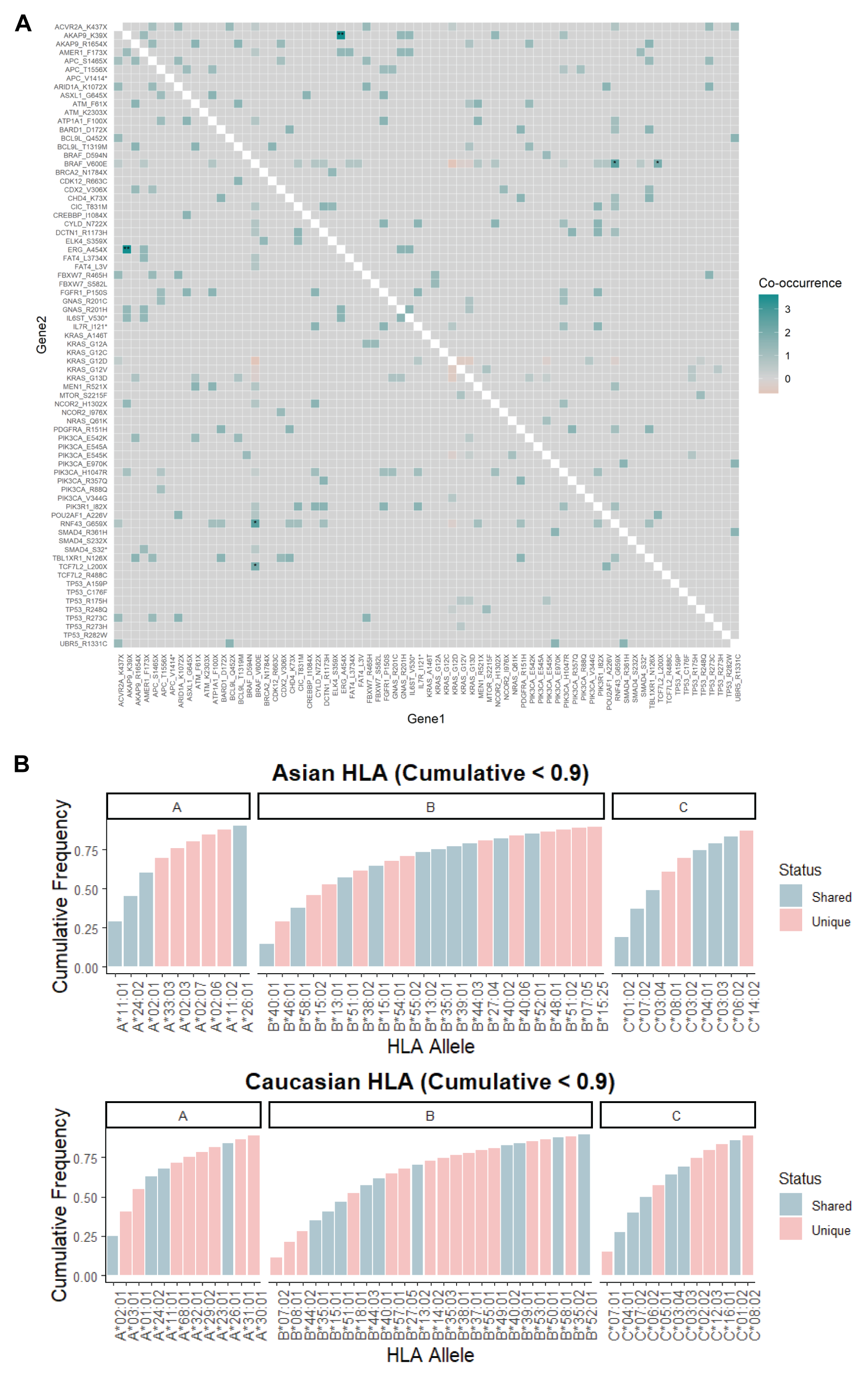

Supplement: Supplementary Figure 1 — Co-mutation landscape and HLA-I allele coverage for the recurrent neoantigen panel. (A) Pairwise co-occurrence and mutual exclusivity matrix for frequently mutated genes in the panel. Statistically significant co-mutations are highlighted in green (***p< 0.001). (B) Top panel: Cumulative frequency of HLA-A, -B, and -C alleles among Asian populations, covering up to 90% cumulative allele frequency. Alleles are color-coded by whether they are shared with (blue) or unique compared to (pink) the Caucasian population. Bottom panel: Same as above, shown for comparison across allele categories and loci. [file Image1.tif]

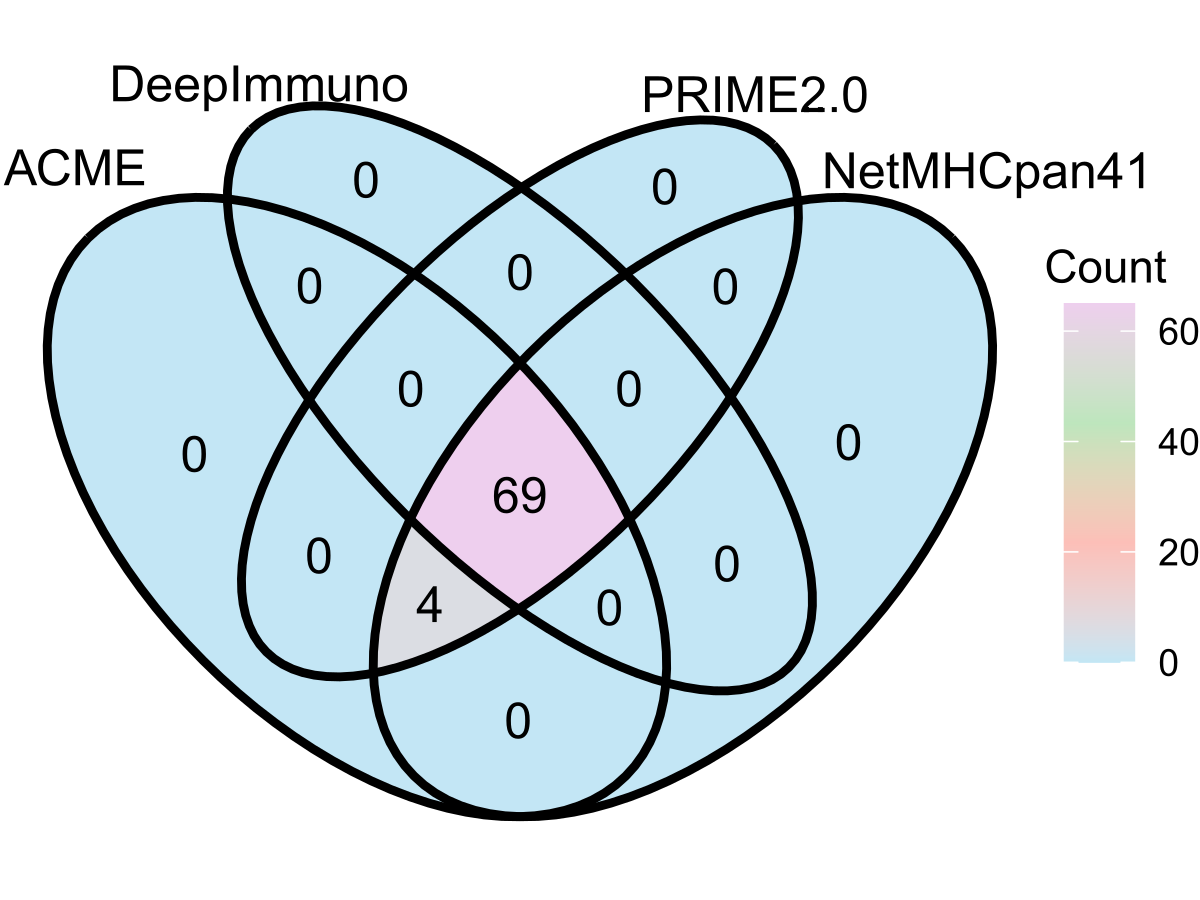

Supplement: Supplementary Figure 2 — Concordance of immunogenicity predictions for neoantigen candidates in the off-the-shelf (OTS) panel across four prediction tools (NetMHCpan-4.1, ACME, PRIME2.0, and DeepImmuno). Neoepitopes were considered positive when the predicted score of the mutant peptide exceeded that of the corresponding wild-type peptide. [file Image2.tiff]

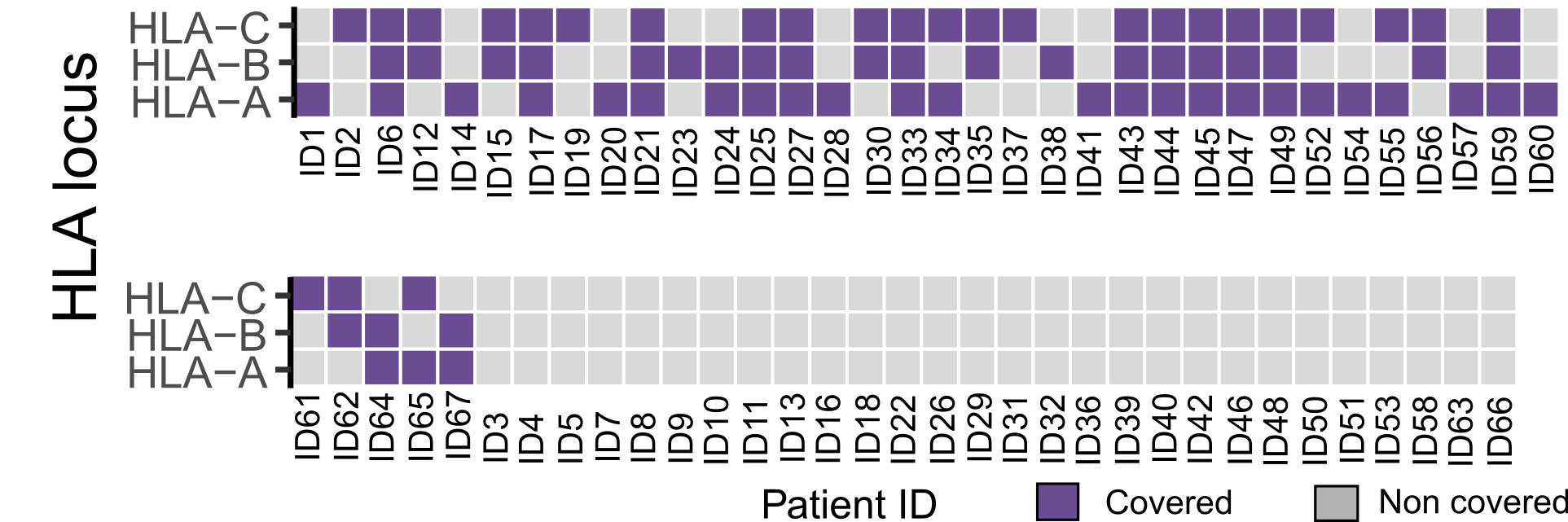

Supplement: Supplementary Figure 3 — HLA class I binding landscape of neoantigen candidates in the off-the-shelf (OTS) panel across the 67 patients in the cohort study. Heatmap showing predicted peptide-HLA class I binding across HLA-A, HLA-B, and HLA-C loci for neoantigen candidates included in the OTS panel across 67 CRC patients. Columns represent individual patients, and rows represent HLA class I loci. Purple squares indicate positive predicted peptide-HLA binding, while grey squares indicate no predicted binding under the applied threshold. [file Image3.tiff]

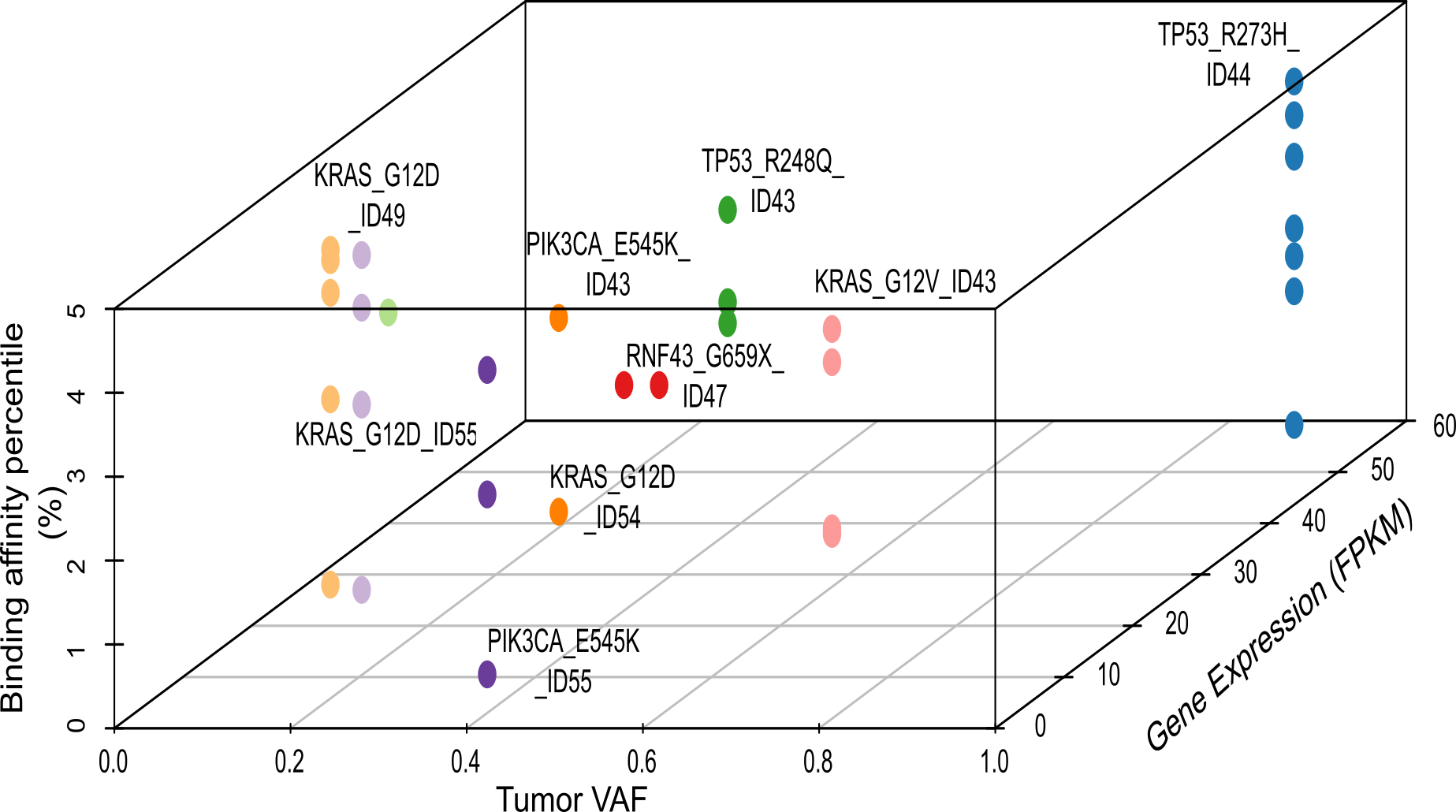

Supplement: Supplementary Figure 4 — Expression level, tumor VAF, and predicted peptide–HLA binding affinity of recurrent mutations in 7 patients included in immunogenicity validation assays. Each point denotes a short peptide derived from the corresponding synthetic long peptide used for in vitro T-cell stimulation. [file Image4.tiff]

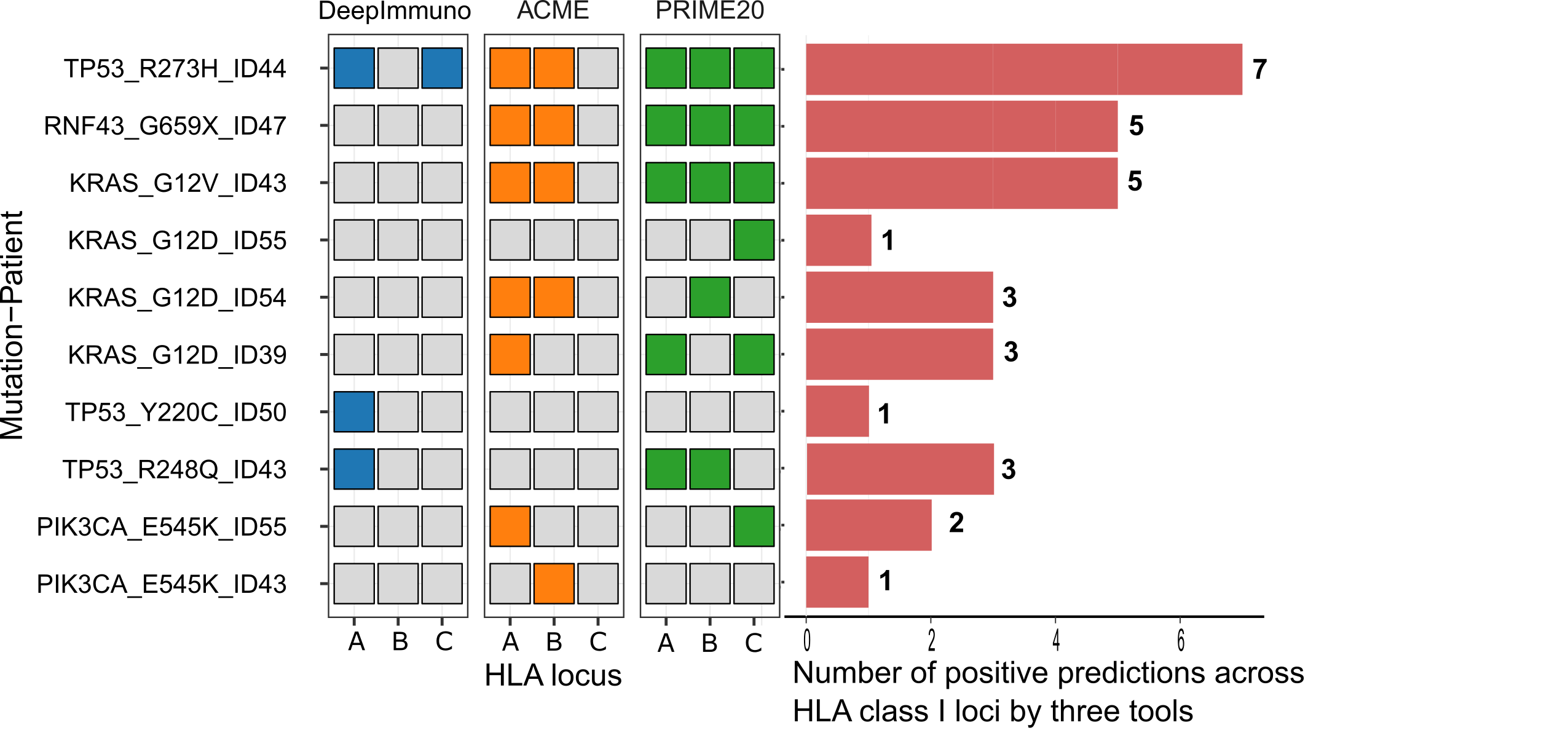

Supplement: Supplementary Figure 5 — Immunogenicity predictions for neoantigens derived from mutations detected in seven patients included in the immunogenicity validation assays. For each prediction tool (DeepImmuno, ACME, and PRIME 2.0), colored squares indicate positive immunogenicity predictions for patient-specific HLA–neoantigen pairs, whereas grey squares indicate negative predictions. A positive prediction was defined as a statistically significant increase (p< 0.05) in immunogenicity scores for mutant-derived short peptide–HLA pairs compared with the corresponding wild-type peptides. [file Image5.tiff]

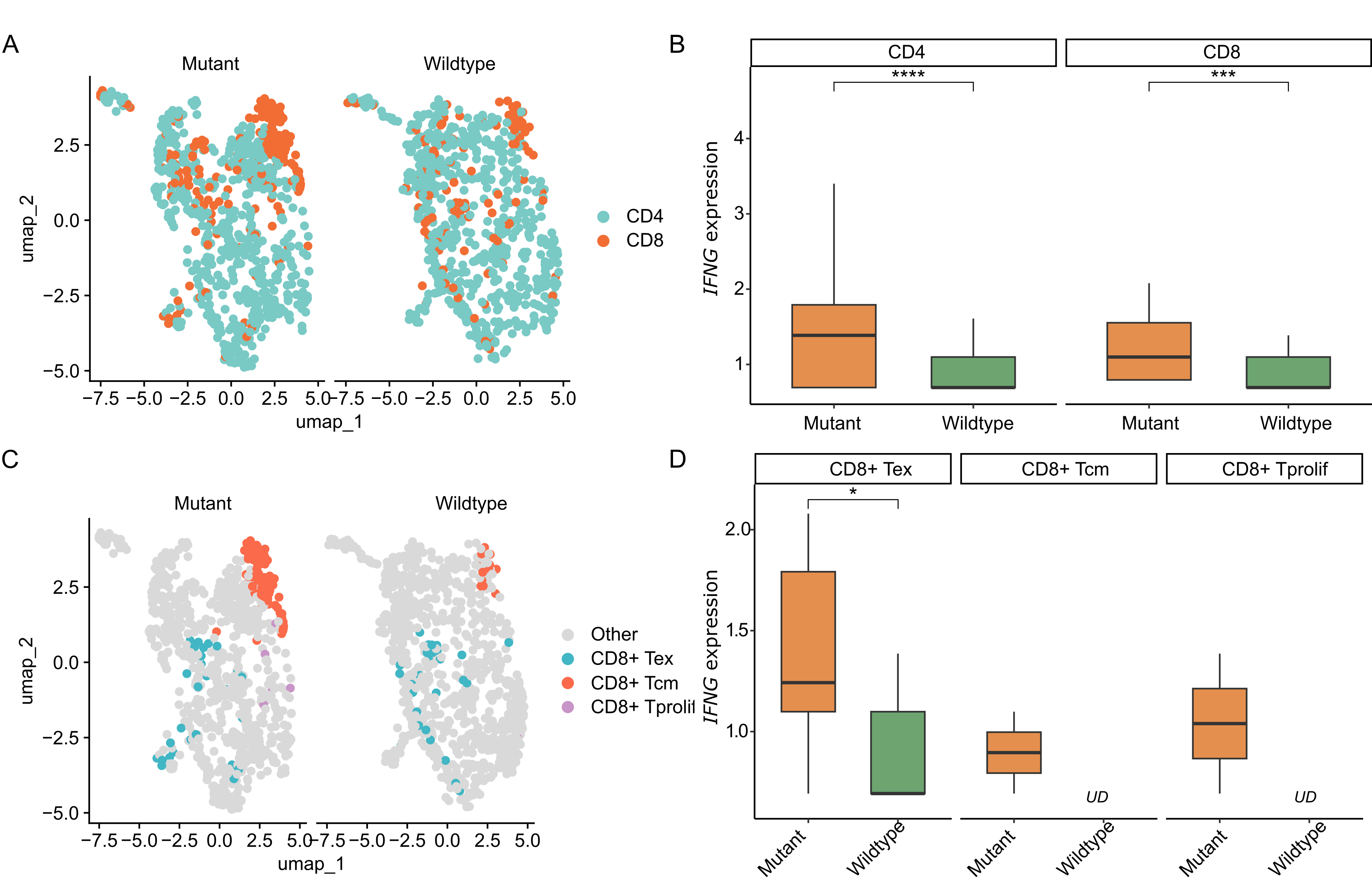

Supplement: Supplementary Figure 6 — Single-cell characterization of neoantigen-reactive T cells following mutant and wild-type peptide stimulation. (A) UMAP visualization of single-cell RNA sequencing data showing CD4+ and CD8+ T-cell populations after stimulation with mutant (left) or corresponding wild-type (right) peptides. Cells are colored by T-cell subset (CD4+, teal; CD8+, orange). (B) Comparison of IFNG expression levels in CD4+ and CD8+ T cells following stimulation with mutant versus wild-type peptides. Box plots show higher IFNG expression in mutant peptide stimulated conditions for both CD4+ and CD8+ T cells, with statistically significant differences indicated (****P< 0.0001; ***P< 0.001). (C) UMAP visualization of single-cell transcriptomes highlighting CD8+ T-cell subsets, including exhausted (CD8+ Tex), central memory–like (CD8+ Tcm), and proliferating (CD8+ Tprolif) populations. Cells are shown separately for mutant and wild-type conditions, with non-CD8 T cells labeled as “Other.” (D) Quantification of IFNG expression levels in CD8+ Tex, CD8+ Tcm, and CD8+ Tprolif subsets comparing mutant and wild-type conditions. Statistical significance was assessed using a two-sided Wilcoxon rank-sum test; *P< 0.05; UD: Undetermined. [file Image6.tiff]

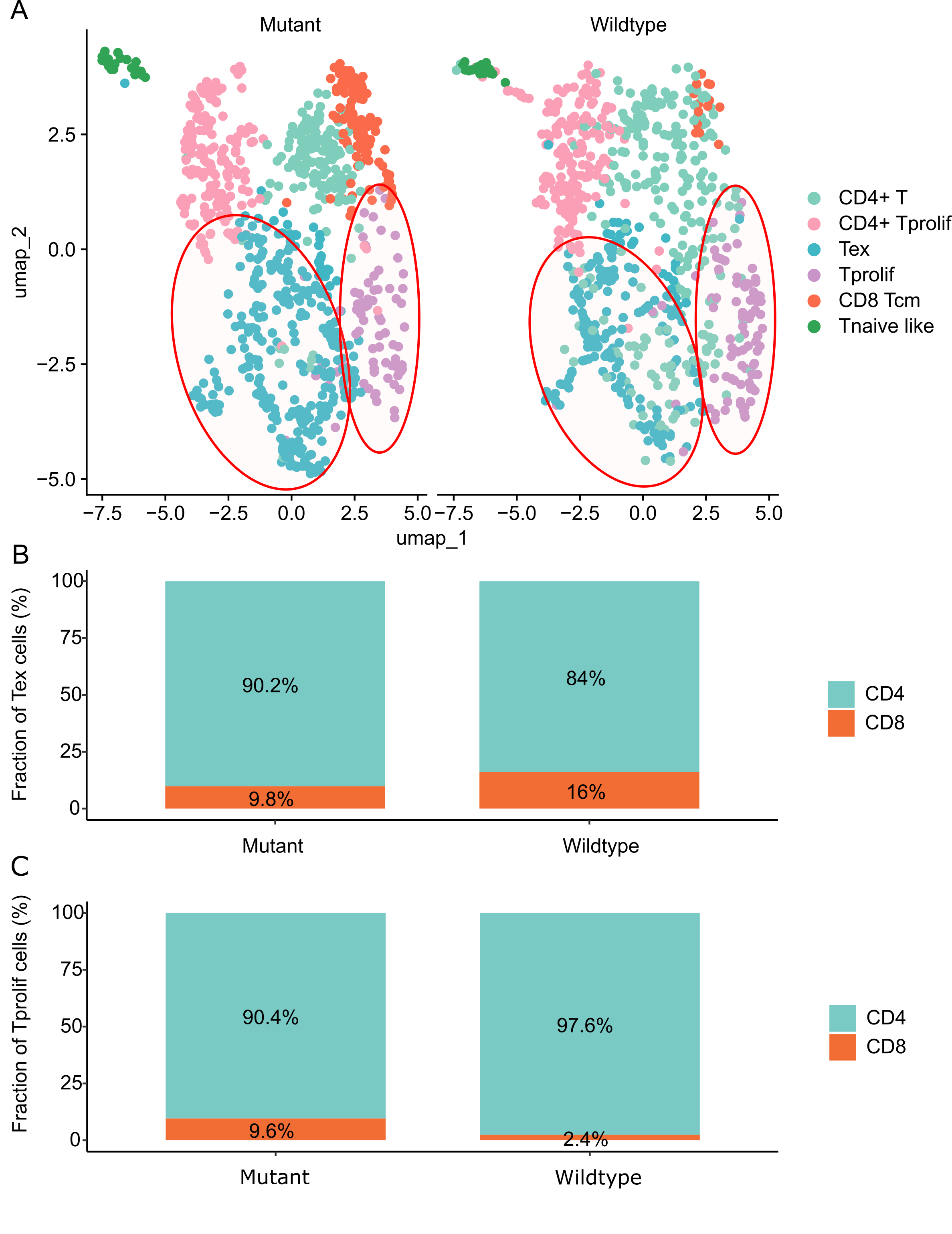

Supplement: Supplementary Figure 7 — Single-cell profiling of neoantigen-reactive T cells following mutant versus wild-type peptide stimulation. (A) UMAP representation of single-cell RNA sequencing data showing T-cell clusters following stimulation with mutant (left) or corresponding wildtype (right) peptides. Distinct T-cell subsets are highlighted, with circled regions indicating exhausted T (Tex) and proliferating T cells (Tprolif) (B) Proportional composition of CD4+ and CD8+ T cells among exhausted T cells (Tex) following mutant and wild-type peptide stimulation. Percentages indicate the relative fraction of each subset. (C) Proportional composition of CD4+ and CD8+ T cells among proliferating T cells (Tprolif) under mutant and wild-type peptide–stimulated conditions. [file Image7.tiff]
